# Editorial Changes

**Published:** 1882-08

**Authors:** 


### Editorial Changes.

Our subscribers will notice that this number of the Journal appears without the names of two of its most able collaborators among its editorial corps, Drs. Howe and Mynter having retired from the management. The Journal will continue under the direction of its present editors. We have assurances that our late associates will favor our readers with frequent contributions from their pen, and their efforts will not be lessened by this separation in advancing the interests of the profession through the medical press. We join in the general expression of regret that circumstances should influence a dissolution of the very pleasant and cordial relations which have existed in the Journal Association since its organization three years ago ; and in parting with our late colleagues, we but express the sentiment of the profession that their labors have largely contributed to the favorable reception given to the Journal under the late management.

This change places increased responsibility and labor upon those who remain; and in our efforts to give the profession a publication worthy of its patronage, we are assured of a large measure of sympathy, through the experience of the past, from our retiring associates.

---

